# Perspectives on West Africa Ebola Virus Disease Outbreak, 2013–2016

**DOI:** 10.3201/eid2206.160021

**Published:** 2016-06

**Authors:** Jessica R. Spengler, Elizabeth D. Ervin, Jonathan S. Towner, Pierre E. Rollin, Stuart T. Nichol

**Affiliations:** Centers for Disease Control and Prevention, Atlanta, Georgia, USA

**Keywords:** Ebola, Ebola hemorrhagic fever, viral hemorrhagic fever, Ebola virus disease, outbreak, emergence, epizootic, spillover, West Africa, viruses, zoonoses

## Abstract

The variety of factors that contributed to the initial undetected spread of Ebola virus disease in West Africa during 2013–2016 and the difficulty controlling the outbreak once the etiology was identified highlight priorities for disease prevention, detection, and response. These factors include occurrence in a region recovering from civil instability and lacking experience with Ebola response; inadequate surveillance, recognition of suspected cases, and Ebola diagnosis; mobile populations and extensive urban transmission; and the community’s insufficient general understanding about the disease. The magnitude of the outbreak was not attributable to a substantial change of the virus. Continued efforts during the outbreak and in preparation for future outbreak response should involve identifying the reservoir, improving in-country detection and response capacity, conducting survivor studies and supporting survivors, engaging in culturally appropriate public education and risk communication, building productive interagency relationships, and continuing support for basic research.

## Abstract 2

Many features of this outbreak reinforce the benefit of continued investment in global health security.

In 1976, the investigation of concurrent outbreaks of a hemorrhagic fever syndrome (Ebola virus disease [EVD]) in Zaire (currently Democratic Republic of Congo) and Sudan (currently Republic of South Sudan) (*1*,*2*) led to isolation of 2 viruses now referred to as Ebola virus (EBOV) and Sudan virus, respectively, and to identification of a newly recognized viral hemorrhagic fever genus, *Ebolavirus* (family *Filoviridae*). Ebolaviruses now include EBOV, Sudan virus, Reston virus, Taï Forest virus and Bundibugyo virus. The other genus in the family *Filoviridae* is *Marburgvirus,* consisting of Marburg virus and Ravn virus (termed marburgviruses; MBGV), both of which are associated with severe disease (Marburg virus disease [MVD]) in humans (*3*,*4*). Before 2013, the largest Ebola outbreak was associated with Sudan virus in Gulu, Uganda, in 2000 that caused 425 cases (224 fatal) (*5*). The largest EVD outbreak associated with EBOV (the same virus responsible for the 2013–2016 outbreak in West Africa) was in Zaire (1976) and caused 318 cases and an associated case-fatality rate of 88% (*2*).

The EVD outbreak in Guinea, Liberia, and Sierra Leone was unprecedented in its sheer magnitude and the emergence of EBOV outside the Congo basin. The effect of the outbreak is profound; as of March 27, 2016, a total of 28,646 EVD cases and 11,323 deaths had been documented (*6*). Furthermore, this outbreak prompted an unparalleled international response: 7 US agencies operated 9 laboratories, and 11 international agencies operated 13 laboratories performing in-country diagnostic tests (Figure 1). The Centers for Disease Control and Prevention (CDC) supported ≈2,300 international deployments of ≈1,600 total personnel (both CDC and non-CDC staff) (*7*); and thousands of personnel from international aid agencies, e.g., World Health Organization, Médecins Sans Frontières, International Rescue Committee, International Finance Corporation, and Public Health England provided in-country support.

**Figure 1:**
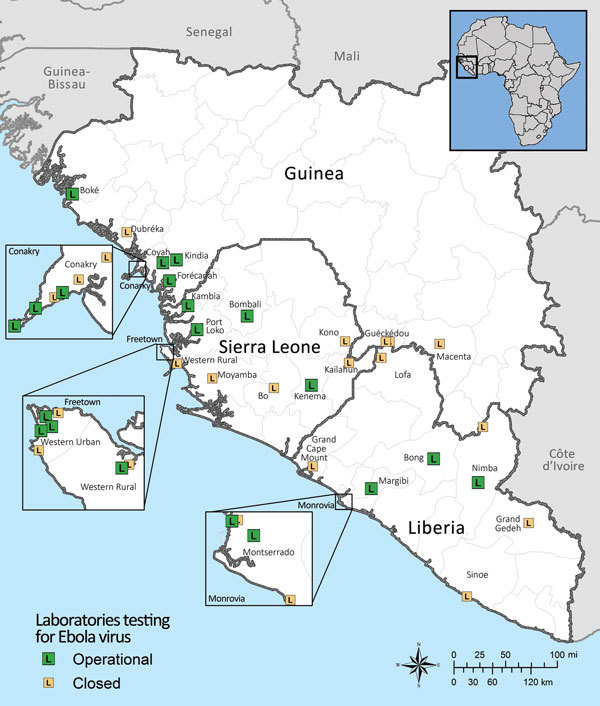
Geographic distribution of diagnostic laboratories currently or previously operational in West Africa during the 2014–2015 Ebola virus response, as of December 9, 2015. Data are from World Health Organization Ebola virus disease situation reports.

The EVD outbreak was not restricted to the 3 heavily affected West African countries; cases also occurred in Senegal, Nigeria, and Mali. In addition, EBOV-infected foreign aid workers were transported for treatment to Europe and the United States, and naturally imported cases (United States, Italy, United Kingdom) and domestic transmission (Spain, United States) were reported for the first time in several countries (*6*). The US EVD response included establishment of EBOV testing in the US Laboratory Response Network. As a result, 57 state, county, and local public health laboratories in 44 states currently are qualified to perform presumptive EBOV real-time quantitative PCR (qPCR).

This EVD outbreak highlights globalization, international social responsibility, and the importance of global health security. As the response to the outbreak progresses, the international research community must continue to address questions of EBOV emergence, pathogenesis, and transmission and advance therapeutic and vaccine development. National and international organizations must critically assess the details of this outbreak and the corresponding response to enable improved response and control of emerging viral outbreaks.

## Why Here? Why Now?

We lack precise answers to these questions. A spillover event is an exceedingly rare but high-consequence event that is likely the most critical initiating factor for an outbreak. Features of the virus phylogenetic analyses and putative reservoir species, and what we know about prior ebolavirus and MBGV spillover events, offer possible insight into “Why here? Why now?” All currently recognized EBOVs appear to share a recent common ancestor ≈50 years ago, probably because of a recent genetic bottleneck (*8*). Current EBOV lineages, first detected in northern Democratic Republic of Congo in 1976, appear to have spread across the Congo basin during this relatively short period and arrived to West Africa only in the past few years. Before the 2013–2016 outbreak, the only definitive evidence of ebolaviruses or the diseases they cause in West Africa was 1 nonfatal human case associated with Taï Forest virus, which caused illness and death in chimpanzees in Côte d’Ivoire in 1994 (*9*).

How EBOV spread across the Congo basin and whether this spread involved movement through bat, nonhuman primate, or other animal populations are unclear. EVD in humans has been linked to preparing and eating nonhuman primate (chimpanzee, gorilla, monkey) or duiker bushmeat (*10*). Contact with bats also has been identified as a putative source of EBOV spillover (*11*). However, the role of bats in virus maintenance and initiation of human disease outbreaks remains unclear. Evidence of bats involvement in the spillover event initiating the 2013–2016 outbreak is limited to anecdotal reports of interactions between bats and villagers in Guinea; no epidemiologic or genetic data associate a putative reservoir species with the current outbreak (*12*). Unlike MBGV, EBOV has yet to be isolated from bats, and no direct evidence links bats to EBOV infection in humans. Regardless, epizootic spillover remains the most widely accepted theory for how the outbreak began.

Experimental infection studies with filoviruses indicate that the viral load in the carcass of an animal that died of EVD would be high (*13*,*14*), but the viral load in the carcass of a healthy reservoir species is probably much lower (*15*,*16*). However, the virus inoculum required to infect animal models of EVD by traditional experimental routes is very low. Thus, viral shedding through excreta or viral load in tissue (eaten or handled raw) of reservoir species might provide sufficient inoculum to initiate virus spillover. In spillover events involving MBGV, sequences from human isolates were ≈99% identical to virus isolates obtained directly from infected bats (*17*); thus, the EBOV spillover event most likely involved little or no virus adaptation. Most EBOV outbreaks appear to involve a single initiating spillover event followed by human-to-human transmission (*18*,*19*), whereas several MVD outbreaks have been associated with multiple spillover events (*4*,*17*). This dissimilarity might reflect a difference in the nature of human interactions with the different primary reservoir species of EBOV and MBGV.

## Why So Big?

Some early speculation about the differences in magnitude between the 2013–2016 EVD outbreak and previous filovirus outbreaks was focused on the presence of a rapidly mutating, highly transmissible or highly virulent EBOV strain. Gire et al. reported a rapid accumulation of interhost and intrahost genetic variation in 99 EBOV genomes from 78 patients in Sierra Leone (*20*). However, later analysis of more EBOV full-length sequences indicated that the overall virus nucleotide substitution rate was consistent with rates observed in previous outbreaks in Central Africa (*8*,*21*). Pathogenesis studies also support that the size of the outbreak and characteristics of EVD in West Africa are not related to change in the virus but instead appear to be a result of factors extrinsic to the virus (*22*–*25*).

Although differences in the outbreak strain do not explain the magnitude of the outbreak, the situation in these West African countries in 2013 made them particularly vulnerable to a large outbreak in the event of the arrival of EBOV or spillover from wildlife. The West African outbreak occurred in an extremely resource-poor area that lacked basic infrastructure and was recovering from the effects of decades of civil war. The consequences of civil instability included the collapse of government institutions and schools, disruption of traditional societal values and structures, poor education standards, and struggling basic healthcare infrastructures (*26*–*28*). Unlike several other African countries, Guinea, Liberia, and Sierra Leone had no past experience in recognizing and managing filovirus outbreaks, and the outbreak occurred in a region with very high endemic levels of malaria that has a similar clinical presentation to EVD. Although these countries had experience with Lassa hemorrhagic fever, that experience most likely negatively affected the initial response: suspecting Lassa might have delayed identifying EBOV and enabled early EBOV transmission. Based on limited chains of human-to-human transmission Lassa virus appears to be less transmissible and requires less stringent use of personal protective equipment and containment to prevent healthcare worker infections.

Slow recognition of suspected cases, inability to accurately diagnose disease, and absence of appropriate surveillance for critical decision-making early in the outbreak severely hampered interruption of EVD spread at key points during the response. Distrust of government and outsiders hindered response efforts, and the spread of conspiracy theories among residents resulted in fear, superstition, and secrecy (*27*). In contrast to prior filovirus outbreak where treatment of cases and transmission occurred in major urban areas (e.g., Kinshasa, Zaire, in 1976; Nairobi, Kenya, in 1980; Kinshasa in 1995; Johannesburg, South Africa, in 1995; Luanda, Angola, in 2005; Kampala, Uganda, 2007; Kampala, Uganda, in 2014 [http://www.cdc.gov/vhf/ebola/outbreaks/history/chronology.html; http://www.cdc.gov/vhf/marburg/resources/outbreak-table.html]); the West African outbreak was the first to include multiple reintroductions to urban areas (such as Conakry) from human cases and extensive urban transmission. Porous borders and high population mobility within each country and into neighboring countries exacerbated widespread dissemination of disease from urban and rural transmission (*27*).

## Future Priorities and Considerations

### Identify the Reservoir

Predicting EBOV epizootics requires increased understanding of virus ecology. Epidemiology, serologic data, and detection of viral RNA support a role of bats and nonhuman primates in EBOV maintenance and spillover transmission from animal reservoirs to humans. However, EBOV has yet to be isolated in nature from any bat species or nonhuman primates. In contrast to EBOV, ecologic and experimental evidence confirms fruit bats (*Rousettus aegyptiacus*) as a reservoir for MBGV, and MBGV spillover events from bats to humans have been documented (*4*,*17*,*29*). Ecologic investigations of *R. aegyptiacus* fruit bats showed seasonal pulses of MBGV spillover events (*30*). MBGV has been isolated from naturally infected *R. aegyptiacus* fruit bats 20 times (*15*,*17*,*31*), and virus replication and oral shedding in the absence of clinical disease was observed in experimentally infected *R. aegyptiacus* fruit bats (*15*).

Although EBOV exhibits ecologic patterns similar to those of MBGV, confirming EBOV reservoir hosts by virus isolation in nature remains elusive. One difficulty in obtaining EBOV isolates from bats, despite many attempts, appears to be identifying and sampling the appropriate bat species. Only 1, or a limited number of, bat species most likely can serve as hosts for each of the filovirus species, a phenomenon also seen with rodentborne hantaviruses and arenaviruses (*32*). In contrast to MBGV, no detectable viremia develops in *R. aegyptiacus* fruit bats experimentally infected with ebolaviruses (Sudan, Ebola, Bundibugyo, Taï Forest, and Reston viruses), and viral RNA detection was localized to the injection site (*33*), suggesting that *R. aegyptiacus* fruit bats are not a competent reservoir species for EBOVs. MBGV ecologic studies support the theory of 1, or a limited number of, host species because infection was found consistently in *R. aegyptiacus* fruit bats but not in *Hipposideros* spp. bats, despite their close interaction (*17*).

**Figure 2:**
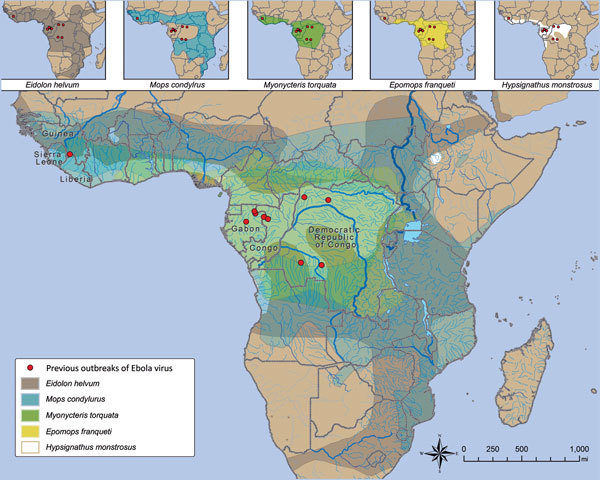
Relationship between location of index case in Ebola virus (*Zaire ebolavirus*) outbreaks and putative reservoir distribution. Ebola virus outbreaks (red dots) and distribution of *Eidolon helvum*, *Mops condylurus*, *Myonycteris torquata*, *Epomops franqueti*, *and Hypsignathus monstrosus* bats (insets) are shown. Data are from the Centers for Disease Control and Prevention and the International Union for the Conservation of Nature.

Although bat species involved in EBOV maintenance have yet to be discovered, limited detection of EBOV RNA and EBOV antibodies has implicated some frugivorous and insectivorous bat species distributed in areas of previous outbreaks, including the little collared fruit bat (*Myonycteris torquata*), hammer-headed bat (*Hypsignathus monstrosus*), Franquet's epauletted fruit bat (*Epomops franqueti*), straw-colored fruit bat (*Eidolon helvum*), and Angolan free-tailed bat (*Mops condylurus*) (*12*,*34*–*36*). Further investigation of these species as putative EBOV reservoirs is warranted; identifying EBOV reservoir species would enable predictive modeling based on distribution (Figure 2) and population dynamics (population size, reproduction, proximity to human populations). Tracking fruit bat migrations across their distribution ranges (Figure 2) including riverine highways and conducting reservoir population surveillance could identify high-risk disease foci before human population exposure, potentially mitigating another spillover and outbreak. In the case of MBGV, understanding the bat reservoir has led to risk reduction measures, such as identifying seasons at high risk for spillover, restricting access of miners or ecotourists to mines and caves with circulating MBGV, and constructing a safe viewing platform at a national park in Uganda.

### Increase In-Country Surveillance, Diagnostic Capacity, and Epidemiologic Support

Curbing disease spread requires rapid identification of the initial human case or cluster after a spillover event to permit patient isolation and timely contact tracing. The payoff for investment in surveillance systems and diagnostic capacity to rapidly identify and respond to outbreaks is illustrated by efforts in Uganda and Democratic Republic of Congo, where the past several EVD and MVD outbreaks were quickly identified and restricted to a few cases. Effective contact tracing to interrupt disease transmission is personnel intensive and requires rapid organization and deployment after notification of suspected cases. In all EVD outbreaks to date, most transmission events involve close-contact human-to-human transmission. Because resources are often limited, case investigations should thoroughly rule out known sources of EBOV transmission before investigating speculated or new (e.g., airborne, environmental, dogs, or asymptomatic human) sources that have never been reported to be associated with disease in previous filovirus outbreaks. Accomplishing the aforementioned recommendations requires increased awareness at the community clinic level, improved quality of central laboratories and capacity for specimen transport (rapid collection and delivery, proper packaging, appropriate storage conditions), and more epidemiologists throughout the region. Finally, laboratorians and epidemiologists must work closely with well-trained clinical and infection control personnel to effectively identify and isolate infectious persons (*37*).

### Test Appropriate Samples and Interpret Evidence-Based Data

Accurate diagnosis requires including EBOV in the differential diagnoses for febrile tropical illness and considering the possibility of co-infection with other frequent tropical diseases (e.g., malaria, typhoid). If appropriate diagnostic samples are not collected, cases might be missed at critical points in the response. Blood is the most sensitive diagnostic specimen early in EVD; oral swabbing, although less invasive, does not offer adequate sensitivity until late in the course of disease, but it is a sensitive and appropriate modality for testing postmortem specimens (*13*,*14*). Diagnostic data are critical in patient management and development of clinical recommendations and discharge policies. Interpretation of diagnostics should consider the sensitivity and specificity of the test and specimen type and what the test is detecting. For instance, qPCR is widely used, but a common mistake in interpreting results is that RNA detection is synonymous with the presence of infectious virus or viral shedding, which is not always the case (*38*). Although detectable viral RNA might indicate shedding, it does not equate with shedding, and this fact must be considered when transmission risk is evaluated on the basis of qPCR.

### Investigate Viral Persistence and Physical and Psychological Sequelae in Survivors

The ≈17,000 survivors from the 2013–2016 EBOV outbreak might face major medical and social challenges after recovering from acute disease. Semen samples from EVD survivors in Kikwit, Democratic Republic of Congo, were positive for EBOV by RT-PCR up to 101 days after illness onset, and 1 sample obtained 82 days after disease onset yielded infectious virus (*19*,*39*). In addition, MBGV was isolated from semen of a convalescent patient and was the source of infection of a contact (*40*; reference *41* [Technical Appendix]). Sexual transmission was also implicated in EBOV transmission recently in Liberia (references *42*,*43* [Technical Appendix]). This outbreak confirmed that EBOV can persist in immune-privileged sites and has highlighted the implications of these findings. EBOV has now been isolated from aqueous humor of the eye (reference *44* [Technical Appendix]), semen (references *17*,*45* [Technical Appendix]), and cerebrospinal fluid (reference *46* [Technical Appendix]) of patients in whom the initial viremia cleared. The details and dynamics of EBOV shedding in body fluids after convalescence remain unclear and need to be investigated further, especially in fluids with higher potential for involvement in transmission events (e.g., semen and amniotic fluid) to clearly define specimens and behaviors with transmission risk.

Although understanding putative disease transmission from convalescent patients is essential to prevent EVD, clinicians and public health professionals also must investigate and address disease sequelae and social stigma associated with EVD in affected populations. Convalescent EVD patients reported arthralgia and myalgia more significantly than control patients during the 1995 Kikwit outbreak (*39*). In addition, 15% of the Kikwit survivors interviewed reported ocular sequelae, including ocular pain, photophobia, hyperlacrimation, and loss of visual acuity; all 4 patients reporting ocular sequelae had uveitis that responded to topical treatment (reference *47* [Technical Appendix]). EVD survivors of the Bundibugyo virus outbreak in Uganda in 2007 also had arthralgia and ocular deficits; hearing loss, neurologic abnormalities, sleep disturbance, memory loss, and various other constitutional symptoms. Chronic health problems also were reported (references *48*,*49* [Technical Appendix]).

In addition to medical burdens associated with recovery, survivors are concurrently dealing with considerable psychological issues of fear, denial, and shame. In severe instances, the social stigma associated with disease can be profound, resulting in abandonment by family and friends (reference *50* [Technical Appendix]).

### Increase Public Education and Risk Communication

The response to an EVD outbreak requires rapid, effective, widespread public education. In addition to increased potential for transmission, a lack of public education and knowledge can contribute to panic, anxiety, and psychosocial trauma; fear and distrust of treatment units and responders, sometimes to the point of violence; and isolation, stigmatization, and community ostracism of survivors and family members of patients (references *51*,*52* [Technical Appendix]). Despite extensive communication efforts during the current EVD outbreak, knowledge and understanding of EVD symptoms remained low, and fear of ill and recovered EVD patients and treatment units persists (reference *52* [Technical Appendix]). To be effective, public education must recognize community-specific risks and concerns. This education must balance culturally appropriate messaging in the context of scientifically founded risk reduction messages to minimize human exposure (references *52*–*54* [Technical Appendix]). Risky behavior must be identified, and messages about risky behavior, prevention strategies, and feasible alternatives must be communicated. Establishing in-country community partners should be integrated to health communication; these partners are often most effective at providing behavioral health education and overcoming language and cultural barriers (reference *54* [Technical Appendix]).

Risk communication can prevent or greatly reduce transmission. EBOV transmission occurs through close contact with symptomatic EVD patients. Familial and social networks play a major role in transmission, particularly through caregivers’ contact with infectious fluids from ill persons at home and in healthcare facilities and through contact with deceased persons during funeral rites. Viral transmission is relatively inefficient compared with other highly infectious agents (reference *55* [Technical Appendix]). An exception is the proposed contribution of EBOV superspreaders (reference *56* [Technical Appendix]): persons who infect disproportionally more secondary contacts. For EBOV, superspreaders fall into 2 categories: biologic superspreaders, who shed more virus, and situational superspreaders, who solely because of circumstances or behavior potentially expose more persons, for example, persons who travel extensively, have occupations that interact closely with many persons (e.g., traditional healers), or deceased patients who had a highly attended funeral. Although biologic superspreaders appear to occur in EVD outbreaks (references *57*,*58* [Technical Appendix]), situational superspreaders more notably elicit transmission events, which successful community education on EVD can greatly reduce.

### Promote Productive Interagency Relationships

Overall outbreak prevention and response will benefit greatly from continuing efforts to develop relationships with nongovernment organizations operating in the region and encouragement of constructive reform of national and international response agencies. We believe the very large and complex nature of the outbreak made communication within and among agencies exceptionally difficult during the outbreak. Frequently, well-intended centralized decision-making did not translate into appropriate application in the field. During the outbreak, partnering among agencies evolved in an effort to improve communication and the outcome of collaborative efforts, but further improvements are possible. Delegating roles among agencies in accordance with their strengths and abilities to acquire the necessary resources for epidemiologic investigations, diagnostics, clinical care, media relations, public education, and logistics might improve efficiency.

### Continue Support for Basic Research

Pathogenesis studies and development of diagnostic tests, therapeutic drugs, and vaccines are the foundation of the public heath response. The international scientific community must continue to prioritize research on EBOV and viral pathogens that have yet to manifest into large outbreaks but have the fundamental characteristics to do so: viruses causing high rates of illness and death that are capable of person-to-person transmission and lack therapeutic drugs, vaccines, and other interventions (e.g., Nipah virus and Crimean-Congo hemorrhagic fever virus). The development and study of new tools toward the end of an outbreak is more likely to be hampered by a lack of patients, as we observed with the EBOV vaccine trials and new diagnostic test evaluations. Thus, future vital research projects should be poised to deploy at the start of new outbreaks, which will require prioritization and substantial regulatory forethought and preparation. However, research projects should not detract from outbreak response. The benefits of research investigations and fundamental response efforts must be balanced appropriately. In response to the outbreak, EBOV researchers worked together in a remarkable effort to advance research and address questions from the field in real time. Interagency collaborations and the open communication of data should continue after the outbreak to develop vaccines and therapeutic drugs and to address key questions on EBOV and other high-consequence, high-containment hemorrhagic fever viruses.

## Conclusions

The large size and long duration of the West Africa EVD outbreak and the resulting enormous national and international response efforts yielded many lessons for improved prevention and control efforts for emerging viral diseases. Although the current outbreak comes to a close and other health crises emerge in the news headlines, we must not forget that many features of this tragic outbreak strongly reinforce the benefit of continued investment in global health security efforts.

**Technical Appendix.** Additional references.

## References

[1] World Health Organization. Ebola haemorrhagic fever in Sudan, 1976. Report of a WHO/International Study Team. Bull World Health Organ. 1978;56:247–70 . 307455 PMC2395561

[2] World Health Organization. Ebola haemorrhagic fever in Zaire, 1976. Report of an international commission. Bull World Health Organ. 1978;56:27193. PMC2395567 307456

[3] Johnson ED, Johnson BK, Silverstein D, Tukei P, Geisbert TW, Sanchez AN, Characterization of a new Marburg virus isolated from a 1987 fatal case in Kenya. Arch Virol Suppl. 1996;11:101–14 . 8800792 10.1007/978-3-7091-7482-1_10

[4] Bausch DG, Nichol ST, Muyembe-Tamfum JJ, Borchert M, Rollin PE, Sleurs H, ; International Scientific and Technical Committee for Marburg Hemorrhagic Fever Control in the Democratic Republic of the Congo. Marburg hemorrhagic fever associated with multiple genetic lineages of virus. N Engl J Med. 2006;355:909–19. 10.1056/NEJMoa051465 16943403

[5] Towner JS, Rollin PE, Bausch DG, Sanchez A, Crary SM, Vincent M, Rapid diagnosis of Ebola hemorrhagic fever by reverse transcription-PCR in an outbreak setting and assessment of patient viral load as a predictor of outcome. J Virol. 2004;78:4330–41. 10.1128/JVI.78.8.4330-4341.2004 15047846 PMC374287

[6] World Health Organization. Ebola situation report. 2016 [cited 2016 Apr 25]. http://apps.who.int/ebola/ebola-situation-reports

[7] Frieden TR, Damon IK. Ebola in West Africa—CDC’s role in epidemic detection, control, and prevention. Emerg Infect Dis. 2015;21:1897–905. 10.3201/eid2111.150949 26484940 PMC4622264

[8] Carroll SA, Towner JS, Sealy TK, McMullan LK, Khristova ML, Burt FJ, Molecular evolution of viruses of the family Filoviridae based on 97 whole-genome sequences. J Virol. 2013;87:2608–16. 10.1128/JVI.03118-12 23255795 PMC3571414

[9] Formenty P, Hatz C, Le Guenno B, Stoll A, Rogenmoser P, Widmer A. Human infection due to Ebola virus, subtype Côte d’Ivoire: clinical and biologic presentation. J Infect Dis. 1999;179(Suppl 1):S48–53. 10.1086/514285 9988164

[10] Leroy EM, Rouquet P, Formenty P, Souquière S, Kilbourne A, Froment J-M, Multiple Ebola virus transmission events and rapid decline of central African wildlife. Science. 2004;303:387–90. 10.1126/science.1092528 14726594

[11] Leroy EM, Epelboin A, Mondonge V, Pourrut X, Gonzalez J-P, Muyembe-Tamfum J-J, Human Ebola outbreak resulting from direct exposure to fruit bats in Luebo, Democratic Republic of Congo, 2007. Vector Borne Zoonotic Dis. 2009;9:723–8. 10.1089/vbz.2008.0167 19323614

[12] Marí Saéz A, Weiss S, Nowak K, Lapeyre V, Zimmermann F, Düx A, Investigating the zoonotic origin of the West African Ebola epidemic. EMBO Mol Med. 2014;7:17–23. 10.15252/emmm.201404792 25550396 PMC4309665

[13] Prescott J, Bushmaker T, Fischer R, Miazgowicz K, Judson S, Munster VJ. Postmortem stability of Ebola virus. Emerg Infect Dis. 2015;21:856–9. 10.3201/eid2105.150041 25897646 PMC4412251

[14] Spengler JR, Chakrabarti AK, Coleman-McCray JD, Martin BE, Nichol ST, Spiropoulou CF, Utility of oral swab sampling for Ebola virus detection in guinea pig model. Emerg Infect Dis. 2015;21:1816–9. 10.3201/eid2110.150840 26401603 PMC4593453

[15] Amman BR, Jones MEB, Sealy TK, Uebelhoer LS, Schuh AJ, Bird BH, Oral shedding of Marburg virus in experimentally infected Egyptian fruit bats (*Rousettus aegyptiacus*). J Wildl Dis. 2015;51:113–24. 10.7589/2014-08-198 25375951 PMC5022530

[16] Paweska JT, Jansen van Vuren P, Masumu J, Leman PA, Grobbelaar AA, Birkhead M, Virological and serological findings in *Rousettus aegyptiacus* experimentally inoculated with vero cells-adapted hogan strain of Marburg virus. PLoS ONE. 2012;7:e45479. 10.1371/journal.pone.0045479 23029039 PMC3444458

[17] Towner JS, Amman BR, Sealy TK, Carroll SA, Comer JA, Kemp A, Isolation of genetically diverse Marburg viruses from Egyptian fruit bats. PLoS Pathog. 2009;5:e1000536. 10.1371/journal.ppat.1000536 19649327 PMC2713404

[18] Ladner JT, Wiley MR, Mate S, Dudas G, Prieto K, Lovett S, Evolution and spread of Ebola virus in Liberia, 2014–2015. Cell Host Microbe. 2015;18:659–69. 10.1016/j.chom.2015.11.008 26651942 PMC4711363

[19] Rodriguez LL, De Roo A, Guimard Y, Trappier SG, Sanchez A, Bressler D, Persistence and genetic stability of Ebola virus during the outbreak in Kikwit, Democratic Republic of the Congo, 1995. J Infect Dis. 1999;179(Suppl 1):S170–6. 10.1086/514291 9988181

[20] Gire SK, Goba A, Andersen KG, Sealfon RS, Park DJ, Kanneh L, Genomic surveillance elucidates Ebola virus origin and transmission during the 2014 outbreak. Science. 2014;345:1369–72. 10.1126/science.1259657 25214632 PMC4431643

[21] Hoenen T, Safronetz D, Groseth A, Wollenberg KR, Koita OA, Diarra B, Virology. Mutation rate and genotype variation of Ebola virus from Mali case sequences. Science. 2015;348:117–9. 10.1126/science.aaa5646 25814067 PMC11045032

[22] Albariño CG, Wiggleton Guerrero L, Lo MK, Nichol ST, Towner JS. Development of a reverse genetics system to generate a recombinant Ebola virus Makona expressing a green fluorescent protein. Virology. 2015;484:259–64. 10.1016/j.virol.2015.06.013 26122472

[23] Dunham EC, Banadyga L, Groseth A, Chiramel AI, Best SM, Ebihara H, Assessing the contribution of interferon antagonism to the virulence of West African Ebola viruses. Nat Commun. 2015;6:8000. 10.1038/ncomms9000 PMC4527089 26242723

[24] Marzi A, Feldmann F, Hanley PW, Scott DP, Günther S, Feldmann H. Delayed disease progression in cynomolgus macaques infected with Ebola virus Makona strain. Emerg Infect Dis. 2015;21:1777–83. 10.3201/eid2110.150259 26402165 PMC4593438

[25] Bird BH, Spengler JR, Chakrabarti AK, Khristova ML, Sealy TK, Coleman-McCray JD, Humanized mouse model of Ebola virus disease mimics immune responses in human disease. J Infect Dis. 2016;213:703–11. 10.1093/infdis/jiv538 26582961 PMC4747627

[26] McPake B, Witter S, Ssali S, Wurie H, Namakula J, Ssengooba F. Ebola in the context of conflict affected states and health systems: case studies of Northern Uganda and Sierra Leone. Confl Health. 2015;9:23. 10.1186/s13031-015-0052-7 26257823 PMC4529686

[27] World Health Organization. One year into the Ebola epidemic: a deadly, tenacious and unforgiving virus [cited 2016 Jan 4]. http://www.who.int/csr/disease/ebola/one-year-report/introduction/en/

[28] Bausch DG, Schwarz L. Outbreak of ebola virus disease in Guinea: where ecology meets economy. PLoS Negl Trop Dis. 2014;8:e3056. 10.1371/journal.pntd.0003056 25079231 PMC4117598

[29] Adjemian J, Farnon EC, Tschioko F, Wamala JF, Byaruhanga E, Bwire GS, Outbreak of Marburg hemorrhagic fever among miners in Kamwenge and Ibanda Districts, Uganda, 2007. J Infect Dis. 2011;204(Suppl 3):S796–9. 10.1093/infdis/jir312 21987753 PMC3203392

[30] Amman BR, Carroll SA, Reed ZD, Sealy TK, Balinandi S, Swanepoel R, Seasonal pulses of Marburg virus circulation in juvenile *Rousettus aegyptiacus* bats coincide with periods of increased risk of human infection. PLoS Pathog. 2012;8:e1002877. 10.1371/journal.ppat.1002877 23055920 PMC3464226

[31] Amman BR, Nyakarahuka L, McElroy AK, Dodd KA, Sealy TK, Schuh AJ, Marburgvirus resurgence in Kitaka Mine bat population after extermination attempts, Uganda. Emerg Infect Dis. 2014;20:1761–4. 10.3201/eid2010.140696 25272104 PMC4193183

[32] Mills JN, Childs JE. Ecologic studies of rodent reservoirs: their relevance for human health. Emerg Infect Dis. 1998;4:529–37. 10.3201/eid0404.980403 9866729 PMC2640244

[33] Jones ME, Schuh AJ, Amman BR, Sealy TK, Zaki SR, Nichol ST, Experimental inoculation of Egyptian rousette bats (*Rousettus aegyptiacus*) with viruses of the *Ebolavirus* and *Marburgvirus* genera. Viruses. 2015;7:3420–42. 10.3390/v7072779 26120867 PMC4517108

[34] Leroy EM, Kumulungui B, Pourrut X, Rouquet P, Hassanin A, Yaba P, Fruit bats as reservoirs of Ebola virus. Nature. 2005;438:575–6. 10.1038/438575a 16319873

[35] Pourrut X, Délicat A, Rollin PE, Ksiazek TG, Gonzalez JP, Leroy EM. Spatial and temporal patterns of Zaire ebolavirus antibody prevalence in the possible reservoir bat species. J Infect Dis. 2007;196(Suppl 2):S176–83. 10.1086/520541 17940947

[36] Ogawa H, Miyamoto H, Nakayama E, Yoshida R, Nakamura I, Sawa H, Seroepidemiological prevalence of multiple species of filoviruses in fruit bats (*Eidolon helvum*) migrating in Africa. J Infect Dis. 2015;212(Suppl 2):S101–8. 10.1093/infdis/jiv063 25786916

[37] Chowell D, Castillo-Chavez C, Krishna S, Qiu X, Anderson KS. Modelling the effect of early detection of Ebola. Lancet Infect Dis. 2015;15:148–9. 10.1016/S1473-3099(14)71084-9 25749063

[38] Spengler JR, McElroy AK, Harmon JR, Ströher U, Nichol ST, Spiropoulou CF. Relationship between Ebola virus real-time quantitative polymerase chain reaction–based threshold cycle value and virus isolation from human plasma. J Infect Dis. 2015;212(Suppl 2):S346–9. 10.1093/infdis/jiv187 25941333 PMC4675930

[39] Rowe AK, Bertolli J, Khan AS, Mukunu R, Muyembe-Tamfum JJ, Bressler D, Clinical, virologic, and immunologic follow-up of convalescent Ebola hemorrhagic fever patients and their household contacts, Kikwit, Democratic Republic of the Congo. Commission de Lutte contre les Epidémies à Kikwit. J Infect Dis. 1999;179(Suppl 1):S28–35. 10.1086/514318 9988162

[40] Martini GA, Schmidt HA. Spermatogenic transmission of the “Marburg virus”. (Causes of “Marburg simian disease”) [in German]. Klin Wochenschr. 1968;46:398–400. 10.1007/BF01734141 4971902

