# Supplementary material for: Perspectives on West Africa Ebola Virus Disease Outbreak, 2013–2016
Source: Emerg Infect Dis. 2016 Jun;22(6):956–63. doi: 10.3201/eid2206.160021 (PMC4880067; doi:10.3201/eid2206.160021)
Supplement: Supplementary file 1 — Technical Appendix. Additional references. [file 16-0021-Techapp-s1.pdf]

Article DOI: http://dx.doi.org/10.32032/eid2206.160021

Perspectives on West Africa Ebola Virus Disease Outbreak, 2013–2016

Technical Appendix

Additional References

41. Smith DH, Johnson BK, Isaacson M, Swanapoel R, Johnson KM, Killey M, et al. Marburg-virus disease in Kenya. Lancet. 1982;1:816–20 http://dx.doi.org/10.1016/S0140-6736(82)91871-2. PubMed

42. Christie A, Davies-Wayne GJ, Cordier-Lassalle T, Blackley DJ, Laney AS, Williams DE, et al.; Centers for Disease Control and Prevention (CDC). Possible sexual transmission of Ebola virus - Liberia, 2015. MMWR Morb Mortal Wkly Rep. 2015;64:479–81. Erratum in: MMWR Morb Mortal Wkly Rep. 2015;64:1180. PubMed

43. Mate SE, Kugelman JR, Nyenswah TG, Ladner JT, Wiley MR, Cordier-Lassalle T, et al. Molecular evidence of sexual transmission of Ebola virus. N Engl J Med. 2015;373:2448–54 http://dx.doi.org/10.1056/NEJMoa1509773. PubMed

44. Varkey JB, Shantha JG, Crozier I, Kraft CS, Lyon GM, Mehta AK, et al. Persistence of Ebola virus in ocular fluid during convalescence. N Engl J Med. 2015;372:2423–7 http://dx.doi.org/10.1056/NEJMoa1500306. PubMed

45. Deen GF, Knust B, Broutet N, Sesay FR, Formenty P, Ross C, et al. Ebola RNA persistence in semen of Ebola virus disease survivors—preliminary report. [Epub ahead of print]. N Engl J Med. 2015. **PMID: 26465681**

46. Gulland A. UK nurse who contracted Ebola is readmitted to hospital. BMJ. 2016;352:i1134 http://dx.doi.org/10.1136/bmj.i1134. PubMed

47. Kibadi K, Mupapa K, Kuvula K, Massamba M, Ndaberey D, Muyembe-Tamfum JJ, et al. Late ophthalmologic manifestations in survivors of the 1995 Ebola virus epidemic in Kikwit, Democratic Republic of the Congo. J Infect Dis. 1999;179(Suppl 1):S13–4 http://dx.doi.org/10.1086/514288. PubMed

48. Clark DV, Kibuuka H, Millard M, Wakabi S, Lukwago L, Taylor A, et al. Long-term sequelae after Ebola virus disease in Bundibugyo, Uganda: a retrospective cohort study. Lancet Infect Dis. 2015;15:905–12 http://dx.doi.org/10.1016/S1473-3099(15)70152-0. PubMed

49. Bausch DG. Sequelae after Ebola virus disease: even when it's over it's not over. Lancet Infect Dis. 2015;15:865–6 http://dx.doi.org/10.1016/S1473-3099(15)70165-9. PubMed

50. De Roo A, Ado B, Rose B, Guimard Y, Fonck K, Colebunders R. Survey among survivors of the 1995 Ebola epidemic in Kikwit, Democratic Republic of Congo: their feelings and experiences. Trop Med Int Health. 1998;3:883–5 http://dx.doi.org/10.1046/j.1365-3156.1998.00322.x. PubMed

51. Tambo E, Ugwu EC, Ngogang JY. Need of surveillance response systems to combat Ebola outbreaks and other emerging infectious diseases in African countries. Infect Dis Poverty. 2014;3:29 http://dx.doi.org/10.1186/2049-9957-3-29. PubMed

52. Kobayashi M, Beer KD, Bjork A, Chatham-Stephens K, Cherry CC, Arzoaquoi S, et al. Community knowledge, attitudes, and practices regarding Ebola virus disease—five counties, Liberia, September–October, 2014. MMWR Morb Mortal Wkly Rep. 2015;64:714–8. PubMed

53. Kilianski A, Evans NG. Effectively communicating the uncertainties surrounding Ebola virus transmission. PLoS Pathog. 2015;11:e1005097 http://dx.doi.org/10.1371/journal.ppat.1005097. PubMed

54. Médecins Sans Frontières. An effective Ebola intervention: the Foya approach [cited 2015 Dec 21]. http://www.msf.org.uk/an-effective-ebola-intervention-the-foya-approach

55. Dowell SF, Mukunu R, Ksiazek TG, Khan AS, Rollin PE, Peters CJ. Transmission of Ebola hemorrhagic fever: a study of risk factors in family members, Kikwit, Democratic Republic of the Congo, 1995. Commission de Lutte contre les Epidémies à Kikwit. J Infect Dis. 1999;179(Suppl 1):S87–91 http://dx.doi.org/10.1086/514284. PubMed

56. Althaus CL. Ebola superspreading. Lancet Infect Dis. 2015;15:507–8 http://dx.doi.org/10.1016/S1473-3099(15)70135-0. PubMed

57. Maganga GD, Kapetshi J, Berthet N, Kebela Ilunga B, Kabange F, Mbala Kingebeni P, et al. Ebola virus disease in the Democratic Republic of Congo. N Engl J Med. 2014;371:2083–91 http://dx.doi.org/10.1056/NEJMoa1411099. PubMed

58. Volz E, Pond S. Phylodynamic analysis of Ebola virus in the 2014 Sierra Leone epidemic. PLoS Curr. 2014;6. pii: ecurrents.outbreaks.6f7025f1271821d4c815385b08f5f80e. **PMID: 25914858**
